# Diagnostic accuracy of B-Mode ultrasound and Hepatorenal Index for graduation of hepatic steatosis in patients with chronic liver disease

**DOI:** 10.1371/journal.pone.0231044

**Published:** 2020-05-01

**Authors:** Golo Petzold, Julian Lasser, Janina Rühl, Sebastian C. B. Bremer, Richard F. Knoop, Volker Ellenrieder, Steffen Kunsch, Albrecht Neesse

**Affiliations:** Department of Gastroenterology and Gastrointestinal Oncology, University Medical Center Goettingen, Goettingen, Germany; Medizinische Fakultat der RWTH Aachen, GERMANY

## Abstract

**Background/Aims:**

The aim of our study was to evaluate the diagnostic accuracy of B-Mode ultrasound and Hepatorenal Index (HRI) by high-end devices for the detection and classification of hepatic steatosis in patients with various causes of chronic liver disease (CLD).

**Methods:**

We retrospectively enrolled patients with CLD who underwent liver biopsy and baseline ultrasound between March 2016 and May 2019. Sonographic graduation of steatosis (0°-III°) using B-Mode criteria and HRI were correlated with the histological graduation (S0 (<5% fat), S1 (≥5–33%), S2 (>33–66%) and S3 (>66%). Interobserver agreement was calculated.

**Results:**

157 patients were evaluated. B-Mode ultrasound had a sensitivity of 75.6% and a specificity of 76.0% to differentiate between steatosis and no steatosis (AUROC 0.758). Using B-Mode criteria for advanced steatosis (≥II°), specificity for presence of histological steatosis was ≥98.7%. For detection of advanced steatosis (≥S2), sensitivity of B-mode criteria was 90.9%. In a subgroup of patients with advanced liver fibrosis, sensitivity of B-mode criteria was 95.0% for detection of advanced steatosis (S≥2). A HRI cut-off-value of 1.46 differentiates between patients with steatosis and patients without steatosis with a sensitivity of 42.7% and a specificity of 90.7% (AUROC 0.680). Interobserver agreement of both B-Mode and HRI was good to excellent.

**Conclusion:**

B-Mode ultrasound using high-end devices is an excellent method to detect advanced steatosis in patients with various CLD. For diagnosis of mild steatosis, modern ultrasound devices may have higher sensitivity but at the expense of specificity. Stage of fibrosis and etiology of CLD seem not to impact on diagnostic accuracy. The additional calculation of HRI seems to have no additional benefit with regard to detect or grade hepatic steatosis in our study population.

## Introduction

Non-alcoholic fatty liver disease (NAFLD) is the most common cause of chronic liver disease in Western countries[1]. The estimated prevalence worldwide is approximately 25%[2]. NAFLD is a generic term that includes two stadiums of one disease: On the one hand, the simple steatosis without significant necroinflammatory injury (non-alcoholic fatty liver (NAFL)) and on the other hand, the steatosis with inflammation and active lesions of hepatocyte injury (non-alcoholic steatohepatitis (NASH)). Patients with NAFL have a risk of up to 30% to develop a NASH[3]. NASH is a progressive disease and patients have a risk of developing fibrosis, cirrhosis and even hepatocellular carcinoma[4]. NAFLD is associated with obesity, diabetes mellitus type 2 and thus with the metabolic syndrome[5]. A recent study identified the degree of steatosis as a risk factor for the development of significant fibrosis in patients with NAFLD[6].

Hepatic steatosis is defined as abnormal (≥5%) accumulation of triglycerides in the liver[7]. Gold standard for the detection of hepatic steatosis is liver biopsy. The common histological classification graduates the steatosis in four degrees according to the percentage of affected hepatocytes: grade 0 (<5%), grade 1 (≥5–33%), grade 2 (>33–66%) and grade 3 (>66%)[7]. However, liver biopsy is invasive, sometimes painful, and has rare but potentially serious complications[8].

Nevertheless, the diagnosis of NAFL is possible with good accuracy in comparison to the gold standard (histology) using imaging studies.

Magnet resonance imaging (MRI) has highly accurate and reproducible diagnostic performance for evaluating NAFLD, and therefore, has been used in many clinical trials as a non-invasive reference of standard method[9]. However, MRI is costly, time consuming and not available everywhere.

Therefore, ultrasound is the most frequently used primary imaging modality for the evaluation of liver disease. The basic sign for steatosis is the increased echogenicity of the liver parenchyma in comparison to the cortex of the right kidney, because intracellular accumulation of fat vacuoles reflects the ultrasound beam. The sensitivity for detecting steatosis using greyscale ultrasound varies between excellent for higher grade of steatosis and poor for mild steatosis[9,10]. Calculation of the hepatorenal index based on B-Mode ultrasound images showed in a few studies excellent diagnostic accuracy even for diagnosis of mild steatosis[11,12] whereas other studies were contradictory[13,14].

Generally, hepatic steatosis is not only seen in NAFLD and alcoholic liver disease (ALD), but also as secondary cause in patients with other liver diseases like Hepatitis C virus infection (HCV) or Wilson disease[15] or with other chronic liver diseases that are not associated with hepatic steatosis per se. For example, concurrent fatty liver is common in HBV-infected patients and an independent risk factor increasing HBV-associated cirrhosis and HCC development[16]. Furthermore, a recent study showed an increased risk for the presence of cirrhosis in patients with autoimmune hepatitis (AIH) and coincidental NAFLD in comparison to patients with AIH without NAFLD[17]. Similar results have been demonstrated in patients with alpha-1 antitrypsin deficiency and additional NAFLD[18,19]. Therefore, particularly in these patients the diagnosis of an additional fatty liver disease is of high clinical relevance.

Only a few studies exist that examined the diagnostic accuracy of steatosis using greyscale ultrasound[10,20–22] and most of them were performed more than 10 years ago, when high-end ultrasound devices were not available yet.

Furthermore, some of these studies only differed between presence or absence of steatosis, although a widely used ultrasound classification for the degree of steatosis exist[23] and all these studies included only patients with NAFLD, HCV or chronic hepatitis B (HBV), whereas patients with less common causes of CLD were not included.

The aim of our study was to evaluate the diagnostic accuracy of greyscale ultrasound, performed by modern high-end ultrasound devices, for the detection and classification of hepatic steatosis using histology as the reference standard in patients with various causes of chronic liver disease. Furthermore, we aimed to evaluate whether calculating of Hepatorenal Index can improve the diagnostic accuracy.

## Materials and methods

We retrospectively enrolled all patients with chronic liver disease undergoing ultrasound-guided random liver biopsy and baseline ultrasound of the liver between March 2016 and May 2019 at the University Medical Center Goettingen, Germany. Before liver biopsy, detailed clinical examination and elaborate blood tests (at least screening for viral hepatitis, autoimmune liver diseases, hemochromatosis, Morbus Wilson and alpha-1 antitrypsin deficiency) were performed in all patients. The Ethics Committee at the University Medical Center Goettingen waived the need for written informed consent from the participants for this retrospective study and approved the study (Registration no. 28/3/18). The study also conformed to the Helsinki Declaration (2013) and local legislation.

### Transcutaneous liver biopsy

Histopathological fat accumulation of the liver served as the reference standard. Liver biopsy was performed using an 18–gauge semi-automatic full core biopsy instrument (BioPince, Argon Medical devices, USA). Following local anesthesia, sampling was carried out in the right lobe either via an intercostal approach or via subcostal approach, or in the left lobe via subcostal approach using permanent sonographic guidance.

All histologic samples were evaluated by experienced pathologists. Patients with biopsy cylinders of less than 15mm length or not representative specimen were excluded from the analysis. Histological grade of steatosis was defined as follows: Grade S0 (<5% affected hepatocytes), grade S1 (≥5–33%), grade S2 (>33–66%) and grade S3 (>66%)[7].

### B-Mode ultrasound

**Fig 1:**
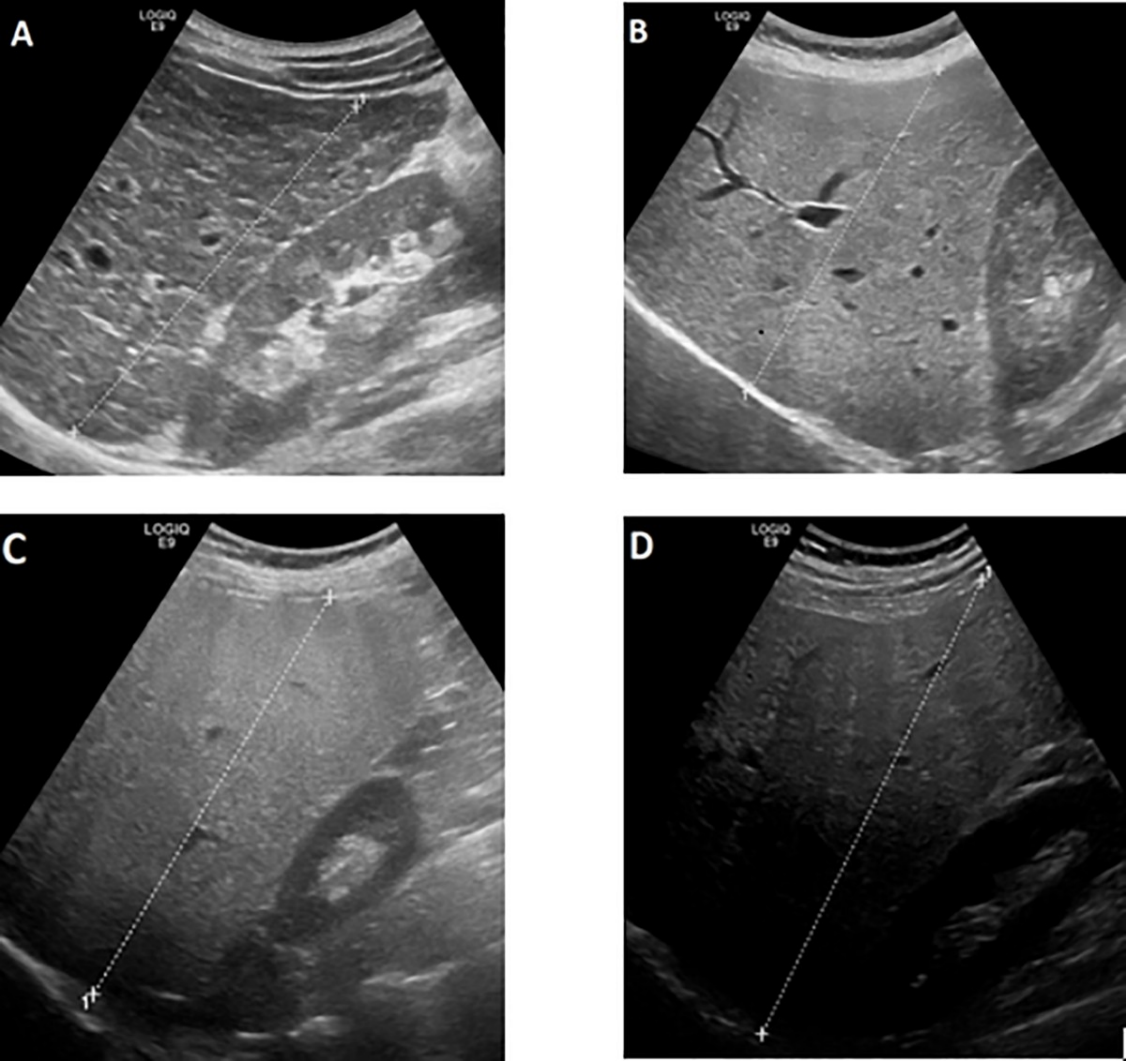
Examples for B-Mode ultrasound steatosis grading 0° (A), I° (B), II° (C) and III° (D).

B-Mode ultrasound of the liver was performed using the high-end ultrasound devices Logiq E9 (GE Medical Systems, Wauwatosa, USA; software R1.0.6) or Hitachi ALOKA (ProSound Alpha 7). An experienced examiner reviewed the stored images. Mandatory requirement for the assessment of steatosis was the appropriate presentation of the right liver lobe, the diaphragm and the right kidney in the same image. Other exclusion criteria were the absence of the right kidney, a hyperechoic cortex of the right kidney due to presence of a chronic kidney disease like Lupus nephritis or the presence of big masses within the right kidney. The classification of steatosis was graded as follows: grade 0 (0°): normal echogenicity of the right liver lobe in comparison with the cortex of the right kidney; grade 1 (I°): slight, diffuse increase in fine echoes in liver parenchyma with normal visualization of diaphragm and intrahepatic vessel borders; grade 2 (II°): moderate, diffuse increase in fine echoes with slightly impaired visualization of intrahepatic vessels and diaphragm; grade 3 (III°): marked increase in fine echoes with poor or nonvisualization of the intrahepatic vessel borders, diaphragm, and posterior right lobe of the liver[23]. Examples of these four sonographic grades are shown in Fig 1. To evaluate interobserver variability, estimation of degree of steatosis was carried out by a second experienced investigator. Results were blinded by both examiners. To analyze the accuracy of B-Mode ultrasound for graduation of hepatic steatosis, we arbitrarily decided to use the results obtained by the first investigator.

### Hepatorenal Index

**Fig 2:**
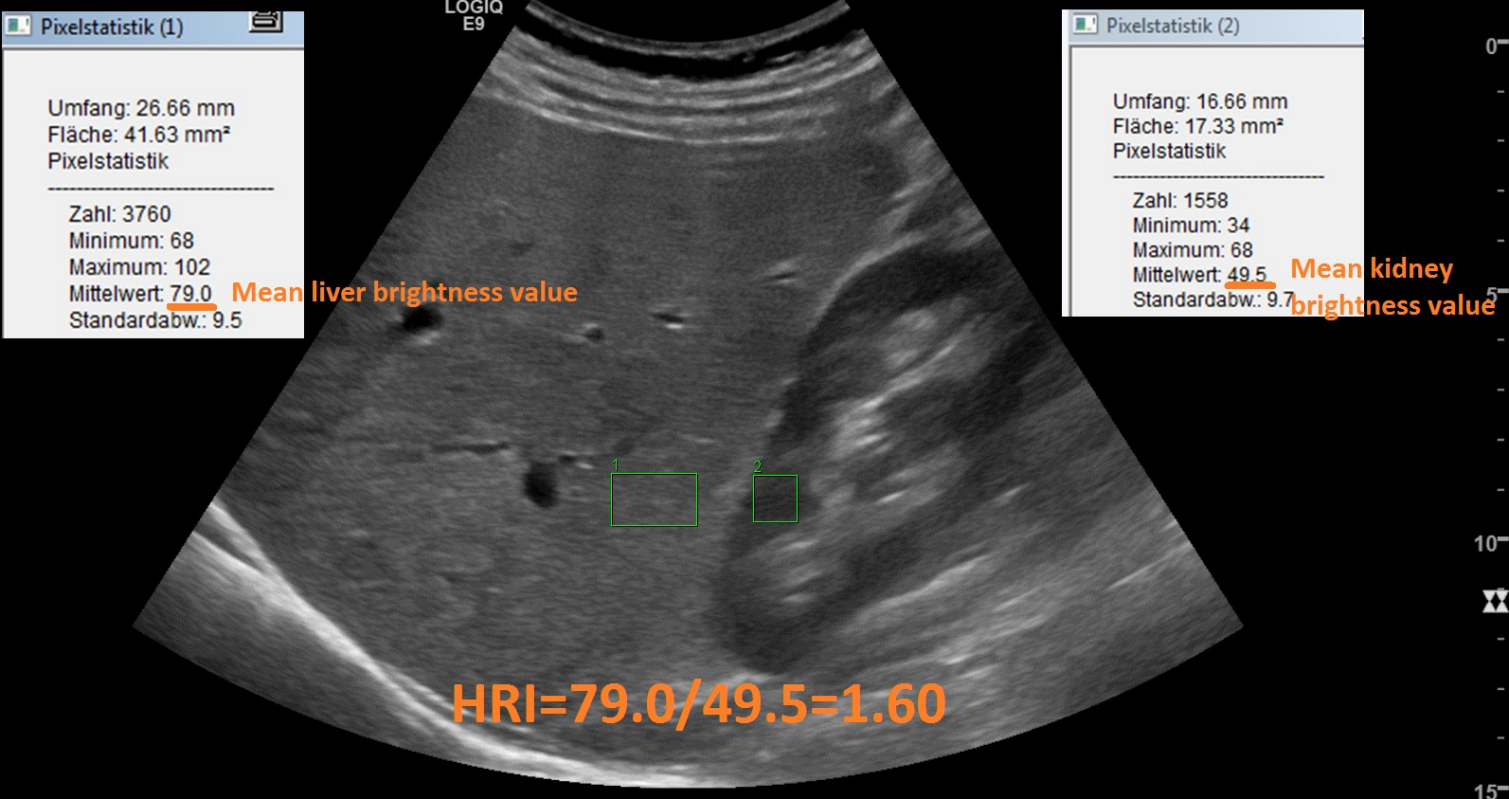
Mean brightness values of the ROI within the kidney (1) and within the liver (2) were automatically demonstrated and HRI was calculated by the formula mean liver brightness value / mean kidney brightness value. Example of a 44-years-old man with primary sclerosing cholangitis (PSC) and mild steatosis.

Additionally, hepatorenal index (HRI) was calculated using a picture archiving and communication system (PACS; GE Healthcare Centricity). Within the recorded images, we chose a region of interest (ROI) free of vessels or artefacts within the liver parenchyma and, in the same depth, within the parenchyma of the cortex of the right kidney, free of fat, large vessels and renal pyramids. The ROI within liver and the ROI within the kidney had to be at least 10mm^2^ in size. Mean brightness value of the ROI was automatically demonstrated and HRI was calculated by the formula mean liver brightness value/ mean kidney brightness value. An example is shown in Fig 2. In the first 30 patients, calculation of HRI was carried out by two investigators with different proficiency. One investigator had performed more than 5000 baseline sonographies; the other investigator had performed approximately 100 supervised baseline sonographies. Results were blinded by both examiners.

### Additional parameters

In addition, we documented additional data: age, gender, body mass index (BMI), current blood-test results (Bilirubin, alanine aminotransferase (ALT), gamma-glutamyltransferase (yGT); not older than 4 weeks, if available), etiology of CLD, presence of diabetes mellitus and histological grade of fibrosis according to Desmet and Scheuer classification[24].

### Statistical analysis

The statistical analysis was performed with SPSS Version 26 (IBM, Armonk, NY, USA). First, we evaluated the normal distribution of quantitative variables. Data were reported as mean including standard deviation. Kruskal-Wallis test followed by Dunn-Bonferroni post hoc test was used to analyze differences of sonographic steatosis (B-Mode and HRI, respectively) between the four cohorts (S0-S3), using the histology results as the reference standard. We defined a statistically significant difference as p <0.05. We used receiver operating characteristic (ROC) curves to calculate the sensitivity and specificity of B-Mode and HRI for the prediction of the presence of steatosis and the different grades of steatosis. The diagnostic performance of B-Mode and HRI was assessed by the area under the receiver operating characteristic curve (AUROC) analysis. Cut-off values of HRI for the prediction of the presence of steatosis grade S≥1, S≥2 and S = 3 were determined as the maximum combined values of sensitivity and specificity (Youden Index). We used Spearman rank method to analyze correlation between grade of steatosis and the parameters HRI, age, gender, Diabetes mellitus, BMI, ALT and Bilirubin. All variables with a p value <0.1 in the univariable analysis were included in the multiple regression analysis model to evaluate independent relation. To evaluate the interobserver reproducibility for HRI, intraclass correlation coefficient (ICC) was obtained and was classified as poor (ICC = 0.0–0.20), fair (0.20–0.40), moderate (ICC = 0.40–0.75) or excellent (ICC> 0.75). Regarding B-Mode criteria, the interobserver agreement percentages were calculated by dividing the number of occasions of complete agreement by the total number of occasions. Additionally, Cohen´s kappa coefficient (κ) was calculated. The kappa statistic was interpreted as follows: less than 0.00, poor agreement; 0.00–0.20, slight agreement; 0.21–0.40, fair agreement; 0.41–0.60, moderate agreement; 0.61–0.80, substantial agreement; and 0.81–1.00, almost perfect agreement[25].

## Results

**Fig 3:**
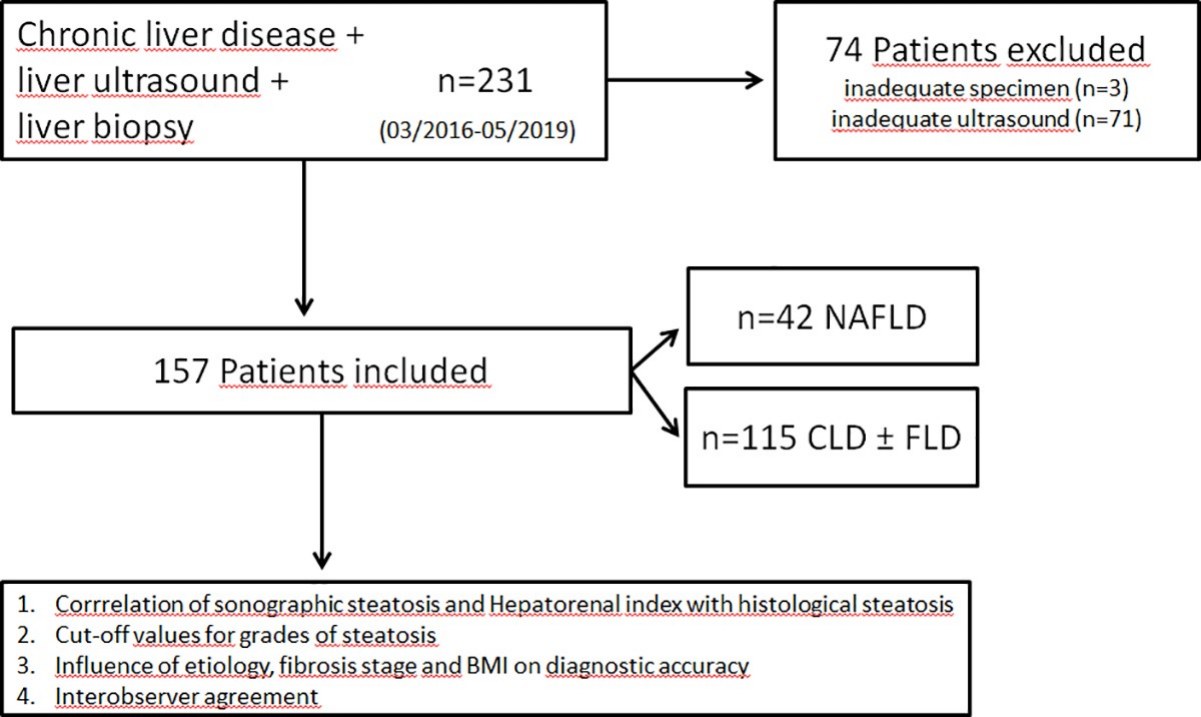
Flow chart of study design. FLD: fatty liver disease.

Altogether liver biopsy was performed in 231 patients with CLD. After applying the exclusion criteria, both adequate biopsy cylinder and an adequate B-Mode image were available in 157 patients. Only these patients were evaluated. The flow chart of the study design is shown in Fig 3. Patients’ characteristics are shown in Table 1. The most common cause of CLD in our cohort was NAFLD (26.8%) followed by AIH (19.1%), ALD, HCV and unknown cause (each 11.5%). Using histology as the reference standard, the following distribution of steatosis grades were detected: 75 (47.8%) patients without steatosis (S0), 38 (24.2%) patients with grade S1, 25 (15.9%) patients with grade S2 and 19 (12.1%) patients with S3.

**Table 1:** Patient characteristics (n = 157).

Characteristic	Mean (±SD) or absolute count
Age	48.23 (±15.29)
Male	70 (44.6%)
Female	87 (55.4%)
BMI (kg/m^2^)	27.66 (±5.55)
<25 kg/m^2^	56
25–30 kg/m^2^	52
>30	49
Diabetes mellitus	32 (20.4%)
Steatosis	82 (52.2%)
I°	38
II°	25
III°	19
Etiology of CLD	
NAFLD	42
AIH	30
ALD	18
HCV	18
Unknown	18
DILI	10
PBC	6
PSC	5
HBV	4
Ischemic Cholangiopathy	3
AAT	2
Hemochromatosis	1

SD: standard deviation; BMI: body mass index; CLD: chronic liver disease; NAFLD: non-alcoholic fatty liver disease; AIH: autoimmune hepatitis; ALD: alcoholic liver disease; HCV: hepatitis C virus infection; DILI: drug induced liver injury; PBC: primary biliary cholangitis; PSC: primary sclerosing cholangitis HBV: hepatitis B virus infection; AAT: alpha-1 antitrypsin deficiency

### B-Mode ultrasound

121 patients were examined using the ultrasound device Logiq E9 (GE), 36 patients were examined using the ultrasound device Hitachi ALOKA. The agreement between the two observers was 89.2% (κ = 0.783) for the presence of steatosis and 80.9% (κ = 0.704) for the grade of steatosis.

Using B-Mode ultrasound criteria, 77 patients had no steatosis (0°), 49 patients had I°, 21 patients II° and 10 patients III°. Using histology as the reference standard, B-Mode ultrasound had a sensitivity of 75.6% and a specificity of 76.0% to differentiate between steatosis and no steatosis. This resulted in a positive predictive value of 0.775 and a negative predictive value of 0.74. AUROC was 0.798 (0.728–0.868). Performing Kruskal-Wallis test and Dunn-Bonferroni post hoc test, significant differences between B-Mode ultrasound grade of patients with S0 and S1 (p = 0.014), S0 and S3 (each p<0.001), S1 and S3 (p<0.001) were observed. A statistical trend was seen between patients with S1 and S2 (p = 0.086) and S2 and S3 (p = 0.090). Using B-Mode ultrasound criteria II° and III°, the specificity for presence of histological steatosis was 98.7% and 100%, respectively. These resulted in positive predictive value of 0.968 and 1.0, respectively. For detection of advanced steatosis (S≥2), sensitivity of B-mode ultrasound criteria was 90.9%.

**Fig 4:**
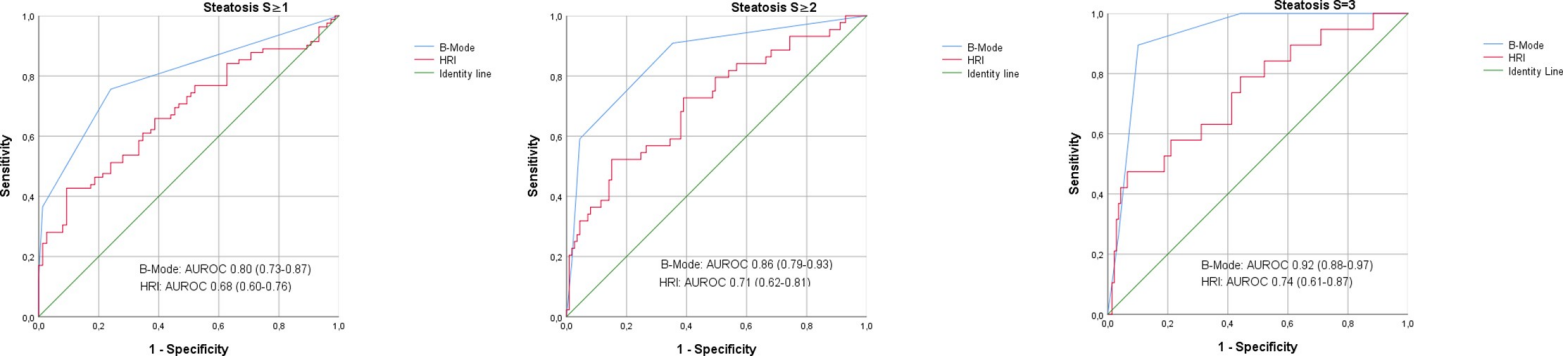
Receiver operator curves (ROC) of B-Mode ultrasound criteria (B-Mode) and Hepatorenal Index (HRI) for the determination of grade of hepatic steatosis.

For steatosis grade S≥2 and S = 3 the best B-Mode cut-off-levels were I° and II°, respectively. AUROC, sensitivity, specificity, positive predictive value and negative predictive value are shown in Table 2. Receiver operating curves are shown in Fig 4.

**Table 2:** B-Mode ultrasound for predicting the grade of steatosis according to optimal cut-off values (n = 157).

Steatosis	AUROC (95% CI)	Cut-off	Sens (%)	Spec (%)	PPV	NPV
**S≥1**	0.798 (0.728–0.868)	I°	75.6	76.0	0.775	0.74
**S≥2**	0.861 (0.793–0.929)	I°	90.9	64.6	0.5	0.948
**S = 3**	0.923 (0.875–0.971)	II°	89.5	89.9	0.55	0.984

AUROC: Area under the receiver operating characteristic curve; Sens: Sensitivity; Spec: Specificity; PPV: Positive predictive value; NPV: Negative predictive value; I°, II°: sonographic grade of steatosis

#### Subgroup of patients without NAFLD

115 patients had another CLD than NAFLD. According to histology results, 75 of them had no steatosis (S0), 24 patients had S1, 11 patients S2 and 5 patients S3. In this subgroup, B-Mode ultrasound has a sensitivity of 65.0% and a specificity of 76.0% to differentiate between patients with steatosis and without steatosis. This resulted in a positive predictive value of 0.591 and a negative predictive value of 0.803. AUROC was 0.731 (0.629–0.833). Using B-Mode ultrasound criteria II° and III°, the specificity for presence of histological steatosis was 98.7% and 100%, respectively. For detection of advanced steatosis (S≥2) sensitivity of B-mode ultrasound criteria was 87.5%.

#### Influence of liver fibrosis on diagnostic accuracy of B-Mode ultrasound

70 patients had histologically confirmed significant liver fibrosis (F≥2) according to Desmet and Scheuer classification. 33 of these patients had no steatosis, 18 patients had S1, 12 patients had S2 and 8 patients had S3. B-Mode ultrasound has a sensitivity of 68.4% and a specificity of 75.0% to differentiate between patients with steatosis and without steatosis. Using B-Mode ultrasound criteria II° and III°, the specificity for presence of histological steatosis was 96.9% and 100%, respectively. For detection of advanced steatosis (S≥2) sensitivity of B-mode ultrasound criteria was 95.0%.

#### Impact of BMI on diagnostic accuracy of B-Mode ultrasound

The mean BMI was 27.66 kg/m^2^ (±5.55). To examine the impact of BMI on diagnostic accuracy of B-Mode ultrasound for detection of hepatic steatosis, we divided the patients into three groups: (I) normal weight (BMI <25 kg/m^2^), (II) overweight (BMI 25–30 kg/m^2^) and (III) obesity (BMI >30kg/m^2^). Sensitivity and specificity of B-Mode ultrasound to differentiate between patients with steatosis and without steatosis were 66.7% and 63.2% (group I), 71.4% and 87.5% (II) and 83.3% and 92.3% (III). These resulted in a positive predictive value and negative predictive value of 0.461 and 0.801 (group I), 0.869 and 0.724 (II) and 0.968 and 0.666 (III).

### Hepatorenal Index (HRI)

HRI was calculated in all 157 patients. To determine interobserver agreement of this method, HRI of the first 30 patients was carried out blinded by two investigators. Using ICC, agreement was excellent (0.934).

**Fig 5:**
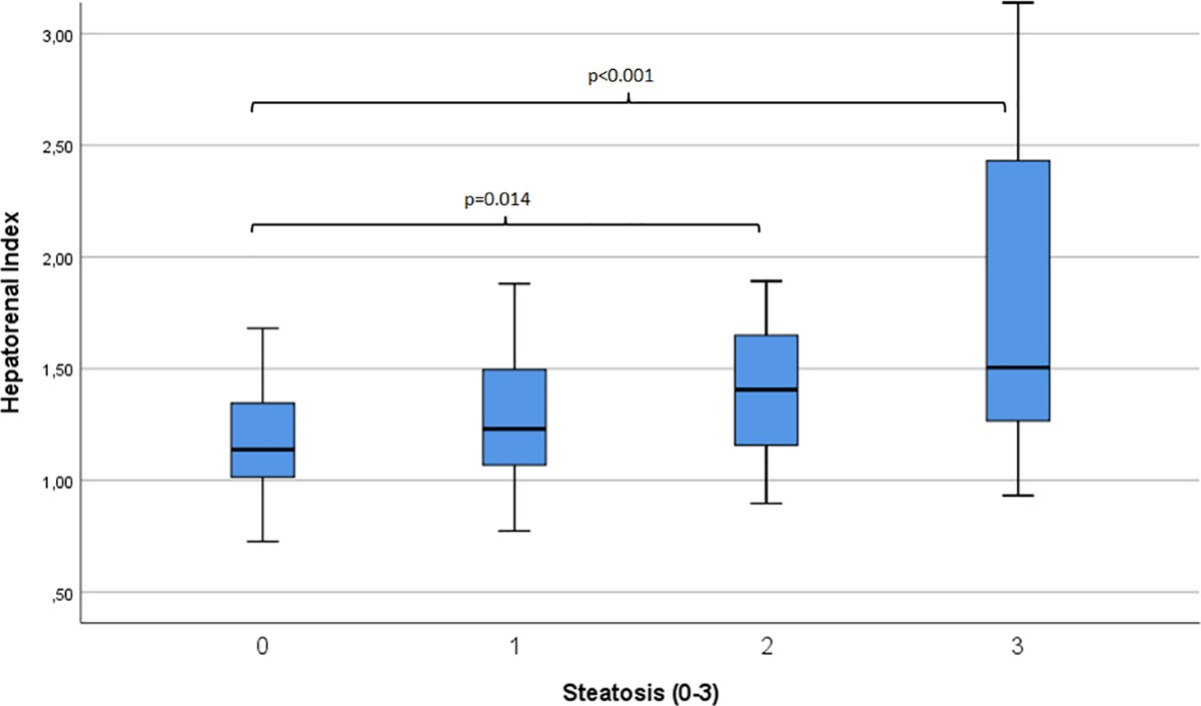
Hepatorenal Index depending on the grade of histological steatosis (n = 157).

Mean size of ROI within the liver was 18.16cm^2^, mean size of ROI within the kidney was 16.72cm^2^. Mean HRI was 1.55 (±1.48). Median HRI values of the four cohorts (S0-S3) are shown in Fig 5 (Boxplot). Performing Kruskal-Wallis test and Dunn-Bonferroni post hoc test, significant differences between HRI of patients with S0 and S3 (p<0.001) and S0 and S2 (p = 0.014) were observed. A statistical trend was seen between patients with S1 and S3 (p = 0.083). No differences were observed between S0 and S1 (p = 0.446), S0 and S2 and S2 and S3 (each p = 1.0).

#### Cut-off values for grades of steatosis

A HRI cut-off-value of 1.46 differentiates between patients with steatosis and patients without steatosis with a sensitivity of 42.7% and a specificity of 90.7%. These resulted in a positive predictive value of 0.834 and a negative predictive value of 0.592. The AUROC was 0.680 (0.597–0.763). A sensitivity of 85.4% was achieved at a HRI-value of 1.05, at the expense of a specificity of 30.7%. The best cut-off values for steatosis grade S≥2 and S = 3 were 1.48 and 1.79, respectively. AUROC, sensitivity, specificity, positive predictive value and negative predictive value are shown in Table 3, receiver operating curves are shown in Fig 4.

**Table 3:** Hepatorenal Index (HRI) for predicting the grade of steatosis according to optimal cut-off values (n = 157).

Steatosis	AUROC (95% CI)	Cut-off	Sens (%)	Spec (%)	PPV	NPV
**S≥1**	0.680 (0.597–0.763)	1.46	42.7	90.7	0.834	0.592
**S≥2**	0.713 (0.620–0.806)	1.48	52.3	85.0	0.576	0.821
**S = 3**	0.737 (0.611–0.863)	1.79	47.4	93.5	0.501	0.928

AUROC: Area under the receiver operating characteristic curve; Sens: Sensitivity; Spec: Specificity; PPV: Positive predictive value; NPV: Negative predictive value

#### Correlation of other parameters with histological grade of steatosis

Performing univariate analysis, the parameters HRI (Spearman’s rho = 0.364; p<0.001), BMI (Spearman’s rho = 0.397; p<0.001) and Diabetes mellitus (Spearman’s rho = 0.262; p = 0.001) revealed a positive significant correlation with grade of steatosis. No correlation was observed between age (p = 0.946), gender (p = 0.211), ALT (p = 0.399), yGT (p = 0.382) and Bilirubin (p = 0.072) and the grade of steatosis. Current ALT, yGT and Bilirubin values were available in 148 of 157 patients. Performing multivariable analysis, only the correlation between HRI (p<0.001) and BMI (p = 0.004) and grade of steatosis remained significant.

## Discussion

We examined the diagnostic accuracy of B-Mode ultrasound, using high-end devices, and HRI for detecting and grading hepatic steatosis in patients with various causes of chronic liver disease with histology as the reference standard. We were able to show that B-Mode ultrasound has excellent sensitivity to detect moderate and severe steatosis. Sensitivity for detecting mild steatosis was slightly lower. In the presence of sonographic criteria of a higher-grade steatosis (i.a. impaired visualization of diaphragm), the specificity of the presence of steatosis was nearly 100%. These results were also seen in the subgroup of patients with other chronic liver diseases than NAFLD. This is of special interest, because the additional presence of steatosis in CLD, especially in patients with chronic hepatitis B virus infection and also in patients with AIH, may be accompanied with increased mortality[16,17]. Therefore, the early detection of additional steatosis is of great importance for the further monitoring and treatment of these patients. Furthermore, we were able to show that presence of significant fibrosis has no marked impact on diagnostic accuracy of hepatic steatosis using B-Mode ultrasound criteria. In contrast, an older study suggested that in patients with chronic HCV and fibrosis the use of B-Mode ultrasound criteria can overestimate the grade of steatosis[20]. As mentioned, in our study sensitivity of ultrasound for mild steatosis was 76%. Taking into account the results of previous studies that were published between the years 2006 and 2010, sensitivity was between 53% and 66%[10,21,22,26]. One possible explanation of the better sensitivity in our study is the use of modern high-end ultrasound devices that can show differences in echogenicity more clearly.

However, this seems to be at the expense of specificity, which was lower in our study (76%) in comparison with the previous studies (77%-93%). Interestingly, sensitivity to detect hepatic steatosis using B-Mode criteria was higher in overweight and obese patients in comparison to normal weight patients. On the other hand, ruling out hepatic steatosis was improved in normal weight patients. These findings may be affected by a higher prevalence of fatty liver in the overweight and obese group.

Using the above mentioned B-Mode criteria for graduation of hepatic steatosis, interobserver agreement in our study was substantial and tended to be better compared with previous studies[25].

The additional calculation of HRI based on the stored B-Mode ultrasound images seems to have no additional benefit with regard to the detection or grade of hepatic steatosis. We were able to show an independent positive correlation between HRI and grade of steatosis, but reliable differentiation between patients without steatosis and patients with mild steatosis was not possible. Diagnostic accuracy using HRI was worse than using B-Mode criteria. Interobserver agreement of the method was excellent. This is in line with previous studies[11]. In consideration of the current literature the diagnostic accuracy of HRI varied widely between different studies, using histology as the reference standard: Sensitivity for mild steatosis varied between 62.5% and 100%[14,13,11,27], specificity reached from 54% to 95%. Optimal cut-off values for mild steatosis has a range from 1.28[27] to 2.01[14]. The different results of all studies examined the accuracy of HRI to detect hepatic steatosis indicate that HRI heavily depends on the used ultrasound device and the cause of liver disease. In our cohort, HRI cut-off values for the graduation of steatosis were very close to each other. A possible explanation of the relatively low HRI values in patients with higher grade of steatosis could be the increased ultrasound beam attenuation. This phenomenon leads to a reduced brightness in the region of interest within the liver.

Furthermore, the lower sensitivity in our study could be explained by the use of two different ultrasound devices. In addition, the enrolled patients in our study represent a broad spectrum of different chronic liver diseases, whereas other studies included mainly patients with NAFLD, HBV or HCV. Further prospective studies performed by different ultrasound devices including patients with uniform chronic liver diseases are needed. Nevertheless, also in our collective HRI was appropriate to rule out moderate or severe steatosis with high probability.

Our study has potential limitations: First, the study was performed as retrospective study with all its known limitations. Second, we used two different ultrasound machines and different physicians performed the B-Mode examinations. Especially crucial parameters like gain setting and frequency were partially different between the stored images. Furthermore, only one liver biopsy was performed depending on the interventional sonographer’s discretion. Indeed, one study in which agreement between paired biopsy specimens in patients with NAFLD was assessed revealed high agreement for steatosis grade[28]. The prevalence of advanced fibrosis in our cohort is quite high and reflects patient cohorts in a tertiary center. This potentially limits the applicability of the findings to low prevalence settings, e.g. for screening purposes in outpatients. However, one aim of our study was to examine the diagnostic accuracy of ultrasound for graduation of hepatic steatosis also in patients with advanced fibrosis.

The strength of our study are the relatively large number of patients under real-world conditions and the inclusion of a broad spectrum of chronic liver diseases at different stages and the presence of histological confirmation as the reference standard in all patients. Furthermore, we evaluated interobserver agreement both for B-Mode ultrasound criteria and for HRI.

In summary, we were able to show that B-Mode ultrasound using high-end devices is an excellent method to detect moderate and severe steatosis not only in patients with NAFLD, but also in patients with various chronic liver diseases at different fibrosis stages. For diagnosis of mild steatosis, modern ultrasound devices may have higher sensitivity but at the expense of specificity. The additional calculation of HRI seems to have no additional benefit with regard to detect or grade hepatic steatosis in our study population. Interobserver agreement both for B-mode criteria and for HRI is good to excellent.

## Supporting information

S1 Data (PDF)
