# Supplementary material for: Diagnostic accuracy of B-Mode ultrasound and Hepatorenal Index for graduation of hepatic steatosis in patients with chronic liver disease
Source: PLoS One. 2020 May 1;15(5):e0231044. doi: 10.1371/journal.pone.0231044 (PMC7194436; doi:10.1371/journal.pone.0231044)
Supplement: S1 Data — (PDF) [file pone.0231044.s001.pdf]

Patient number	sex female=0, male=1	BMI (kg/m²)	Sonographic steatosis (0-3) Reviewer 1	Sonographic steatosis (0-3) Reviewer 2	HRI Reviewer 1	HRI Reviewer 2
1	0	28.4078773	0	0	1.58	1.22
2	0	18.920068	0	0	1.15	1.37
3	0	32.5963719	2	2	1.04	1.04
4	0	28.8754401	2	2	1.25	1.24
5	0	23.3843537	0	0	1.16	1
6	1	26.122449	0	0	1.1	1.12
7	1	23.29123	1	1	1.3	1.35
8	1	21.1007053	0	1	1.19	1.24
9	0	31.245237	0	0	0.99	0.99
10	0	29.7210791	0	0	1.37	1.51
11	0	34.8993764	1	1	1.22	1.08
12	1	30.2992046	2	2	1.48	1.43
13	0	24.8409801	1	1	1.52	1.52
14	1	30.8274743	0	0	1.44	0.86
15	1	22.6346763	1	1	1.43	1.47
16	0	29.3723974	0	1	1.37	1.46
17	0	35.15625	0	0	1.24	1.16
18	1	27.4716889	2	3	3.34	3.85
19	1	21.4619366	1	1	1.58	1.59
20	0	23.1472551	1	1	1.43	1.36
21	1	27.7742709	1	1	1.55	1.5
22	1	30.3405386	0	0	1.14	1.12
23	0	45.5463728	2	2	1.06	1.18
24	1	26.5432099	0	0	1.11	0.92
25	1	27.7388544	3	2	2.15	2.11
26	0	40.6488765	0	0	0.73	0.89
27	1	24.4418021	0	0	0.9	0.9
28	1	26.8809517	0	0	0.82	0.74
29	0	22.405877	0	0	1.19	1.01
30	0	37.4636742	2	2	1.32	1.16
31	1	27.7447483	0	1	1.65	
32	0	24.5089458	0	0	1.35	
33	0	27.8851874	2	2	4.53	
34	0	18.4240363	1	1	1.42	
35	0	35.15625	1	1	1.36	
36	0	26.9896194	0	0	1.06	
37	1	27.2106544	1	1	0.85	
38	1	22.7244035	1	1	1.34	
39	1	21.6049383	0	0	1.1	
40	1	25.8264463	2	2	1.52	
41	0	22.89282	1	1	1.3	
42	0	34.2935528	2	1	2.39	
43	0	32.449973	0	0	0.92	
44	0	33.9100346	2	2	1.5	
45	0	19.2335607	1	0	1.5	
46	1	26.7036236	0	0	1.05	

47	1	20.0617284	0	0	1.06
48	0	26.6727633	1	1	1.54
49	0	19.1570881	0	0	0.94
50	0	19.4734066	0	1	1.12
51	0	19.1000918	0	1	1.31
52	0	35.0765306	0	0	1.1
53	0	17.2248037	0	0	0.93
54	1	20.8307032	0	0	1.15
55	1	26.7299275	3	2	9.93
56	0	23.4235574	1	1	1.78
57	0	27.9680161	0	0	1.05
58	1	20.8327623	0	0	0.95
59	0	21.7079234	0	0	1.1
60	1	21.1463199	0	0	0.77
61	0	24.3374797	1	1	1.92
62	1	29.0123457	0	0	0.89
63	0	30.0432623	0	0	0.99
64	0	37.5	0	1	1.08
65	0	30.7809074	1	1	1.16
66	0	37.7229081	3	3	0.9
67	1	26.794938	0	0	0.94
68	1	28.7037037	1	1	1.13
69	1	36.802686	1	0	1.16
70	1	24.0929705	0	0	1.25
71	1	24.2214533	2	0	1.01
72	1	23.6614386	1	1	1.14
73	1	29.4107589	0	0	0.85
74	1	30.6425357	2	1	1.09
75	0	22.0688094	1	1	1.26
76	0	17.2635445	1	1	1.21
77	1	32.0385784	1	1	1.56
78	1	24.9680097	2	2	1.27
79	0	30.4866574	0	0	1.32
80	1	24.6770213	0	0	1.04
81	0	31.9602273	1	1	1.59
82	0	34.1836735	2	2	1.49
83	1	34.0866052	0	0	0.93
84	0	21.3382106	0	0	1.15
85	0	20.0777479	0	0	1.05
86	0	32.421875	1	1	1.74
87	0	28.3195592	1	1	1.36
88	1	36.8979592	1	1	1.88
89	0	19.3771626	0	0	1
90	1	24.4418021	1	3	0.91
91	1	30.2188578	0	0	1.23
92	1	32.8719723	2	2	3.99
93	1	38.2956573	1	1	1.41
94	0	40.0368481	2	2	4.93
95	1	22.5914381	0	0	0.95
96	1	34.3679138	0	0	1.28

97	1	23.6712536	0	0	0.87
98	0	23.7228107	1	1	1.27
99	0	32.9506803	1	1	1.07
100	1	28.6287924	0	0	1.54
101	1	29.772228	0	0	1.14
102	0	29.7370443	1	2	1.27
103	1	30.026595	0	1	1.35
104	0	26.4380708	1	1	2.38
105	1	32.5259516	1	1	3.69
106	1	25.2160494	0	1	1.04
107	1	28.3265804	0	0	1.09
108	0	23.4375	0	1	1.43
109	0	21.8299522	0	0	1.1
110	0	27.700831	0	0	1.03
111	1	27.7274785	0	0	1.62
112	0	30.859375	2	2	0.93
113	0	27.6360544	3	3	2.06
114	1	27.7574872	0	0	1.49
115	1	33.0168006	1	1	1.47
116	1	26.8554688	1	1	1.76
117	0	35.6513379	3	3	1.65
118	0	21.5138585	1	1	1.68
119	0	22.6940189	1	1	1.41
120	1	38.8723634	2	2	1.26
121	0	32.4584127	0	0	1.07
122	0	27.7447483	0	0	1.03
123	0	28.2283865	1	0	1.12
124	1	27.7742709	1	1	1.22
125	0	28.3937263	0	0	1.32
126	0	26.346494	0	0	1.06
127	0	26.171875	2	1	1.8
128	0	30.3871053	1	1	1.42
129	1	32.488629	3	2	2.48
130	0	24.2214533	1	1	1.45
131	0	26.2917238	2	1	9.09
132	0	20.7612457	2	1	1.17
133	0	23.0517549	1	1	1.21
134	0	31.2213039	0	1	1.05
135	1	35.1027136	3	3	14.05
136	0	20.9643606	1	1	1.4
137	0	24.0929705	0	0	1.03
138	0	27.7767061	0	0	1.66
139	0	30.1193756	0	0	0.92
140	0	20.0692042	0	0	0.99
141	1	24.9337205	0	0	0.93
142	1	31.6373967	3	2	1.37
143	0	26.5118041	0	0	0.89
144	1	31.4056134	0	0	1.13
145	0	22.8623685	0	1	1.15
146	1	29.0858726	3	3	2.35

147	1	22.2222222	0	1	1.11
148	1	28.9978938	1	1	1.44
149	1	27.444545	0	0	0.94
150	1	49.6326531	3	3	1.28
151	1	23.5898355	1	3	1.89
152	1	25.306932	0	0	0.96
153	0	25.5102041	0	0	0.9
154	0	29.0317898	1	1	1.46
155	0	25.3934311	0	1	1.38
156	0	27.4348422	1	1	1.26
157	0	24.419279	1	1	1.18

Device (1=Logiq E9; 0=Hitachi Aloka)	Etiology of CLD	Diabetes mellitus 1=yes, 0=no	Fibrosis (0-4) Desmet	Fat: Affected hepatocyts (%)	Histological steatosis (0-3)	Bilirubin (mg/dl)
1	AIH	0	2	0	0	0.4
1	PBC	0	1	0	0	0.7
0	ALD	0	1	50	2	0.6
1	ALD	0	3	0	0	3
0	unknown	0	1	0	0	1.4
1	ALD	0	0	0	0	0.5
1	unknown	0	0	0	0	0.4
1	PSC	0	2	0	0	0.5
1	unknown	0	4	0	0	0.5
1	DILI	0	1	0	0	3.1
1	NAFLD	0	0	30	1	0.4
0	AIH	0	1	80	3	1.2
1	HCV	0	3	40	2	0.9
1	unknown	0	1	0	0	0.3
1	unknown	0	0	2	0	0.8
1	AIH	0	1	0	0	0.8
1	HCV	0	4	15	1	0.9
1	NAFLD	0	1	60	2	1
1	PSC	0	2	0	0	0.3
1	HCV	1	1	2	0	0.7
1	NAFLD	0	0	20	1	0.6
1	ALD	0	0	25	1	1
1	NAFLD	1	1	90	3	0.2
1	AIH	0	1	0	0	0.6
0	HBV	0	2	20	1	1.6
1	DILI	0	1	0	0	1.3
1	Ischemic Chol	0	1	5	1	14.9
1	unknown	0	0	3	0	
1	PSC	0	0	0	0	1.1
1	NAFLD	1	0	50	2	
1	HCV	0	2	10	1	1
1	HCV	0	2	3	0	0.9
1	NAFLD	0	0	60	2	0.4
0	DILI	0	4	0	0	7.7
0	HCV	0	2	20	1	0.7
1	AIH	0	1	0	0	3.6
0	NAFLD	0	0	25	1	2.1
0	unknown	0	0	3	0	0.4
1	AIH	0	0	0	0	0.5
1	ALD	0	1	60	2	0.7
1	unknown	0	4	0	0	2
1	NAFLD	0	1	80	3	0.4
0	AIH	0	2	0	0	7.5
0	NAFLD	1	1	80	3	0.6
1	AAT	0	0	20	1	0.5
1	HCV	0	2	20	1	0.8

1	HCV	1	2	30	1	0.9
0	AIH	0	2	0	0	0.2
0	PSC	0	0	0	0	0.8
0	DILI	0	3	20	1	0.3
0	AIH	0	3	0	0	0.5
1	AIH	0	0	0	0	9.6
0	DILI	0	0	0	0	13.7
1	HCV	0	4	30	1	1.9
1	NAFLD	1	0	15	1	0.7
1	NAFLD	0	0	40	2	0.3
0	unknown	0	0	0	0	0.6
1	AIH	0	4	0	0	1.5
1	DILI	0	1	0	0	0.9
1	ALD	1	3	5	1	0.4
0	AIH	0	4	0	0	3.3
1	PSC	0	3	0	0	1.3
1	ALD	1	0	0	0	0.4
1	AIH	1	2	5	1	1
0	NAFLD	0	0	40	2	0.4
1	NAFLD	1	2	60	2	0.5
0	unknown	0	4	0	0	0.6
1	NAFLD	1	0	30	1	2.1
1	HCV	1	3	40	2	0.9
1	AIH	0	2	0	0	0.7
0	Ischemic Chol	0	0	20	1	0.8
1	HCV	0	2	60	2	0.5
0	HCV	0	3	0	0	1.4
1	HCV	0	2	20	1	0.6
0	AIH	0	0	0	0	0.4
0	ALD	1	1	20	1	0.9
1	NAFLD	0	3	60	2	
0	HCV	0	3	70	3	
1	AIH	0	3	0	0	0.5
1	HBV	1	4	0	0	0.6
0	DILI	1	2	0	0	1.8
1	NAFLD	1	2	66	2	0.7
1	NAFLD	1	4	30	1	0.7
1	AAT	0	4	0	0	0.6
0	Ischemic Chol	0	1	0	0	13.1
1	NAFLD	1	3	30	1	
1	NAFLD	0	0	30	1	1.4
1	HCV	0	4	30	1	
1	HCV	1	2	1	0	0.4
0	ALD	0	4	60	2	2.6
1	HCV	1	1	0	0	
1	NAFLD	0	1	80	3	0.4
1	NAFLD	1	4	50	2	0.9
1	ALD	0	4	80	3	5.6
0	unknown	0	0	0	0	0.7
1	ALD	1	1	1	0	

1	NAFLD	0	0	20	1	0.8
0	NAFLD	0	2	40	2	1
0	NAFLD	1	0	30	1	0.6
1	HBV	0	2	30	1	0.5
1	ALD	0	3	1	0	1.1
0	HCV	0	3	50	2	
1	NAFLD	0	3	5	1	0.8
1	AIH	0	1	10	1	0.5
1	NAFLD	1	3	70	3	0.5
0	ALD	0	0	60	2	1.1
0	ALD	0	4	0	0	1.2
1	PBC	0	1	0	0	0.4
1	AIH	0	3	0	0	1.6
0	AIH	0	1	0	0	1.1
1	AIH	0	2	0	0	1.7
1	NAFLD	1	0	80	3	0.5
1	NAFLD	1	3	70	3	1.3
1	NAFLD	0	2	50	2	4.5
0	NAFLD	0	0	10	1	1
1	DILI	0	0	20	1	0.8
1	NAFLD	1	0	60	2	0.3
1	PBC	0	1	0	0	0.4
1	AIH	1	2	30	1	1
1	NAFLD	1	2	80	3	0.3
1	NAFLD	0	1	50	2	1.1
1	AIH	0	3	0	0	1.4
1	ALD	0	4	80	3	0.7
1	NAFLD	0	0	20	1	1
1	AIH	0	0	10	1	1.9
1	PBC	0	1	0	0	0.6
1	NAFLD	0	0	80	3	1.3
1	DILI	0	3	20	1	3.7
1	NAFLD	0	3	90	3	0.8
1	unknown	0	2	0	0	0.5
1	NAFLD	0	0	80	3	0.4
0	ALD	0	3	80	3	3.2
1	unknown	0	1	0	0	1.5
1	AIH	0	0	0	0	0.5
1	ALD	1	4	60	2	1
1	AIH	0	1	0	0	0.7
1	AIH	0	3	0	0	1.2
1	AIH	0	0	40	2	0.3
1	PBC	0	2	0	0	0.8
1	AIH	0	2	0	0	0.5
1	Hemochromat	0	0	0	0	1.4
1	NAFLD	0	0	80	3	0.5
1	unknwon	0	0	0	0	0.6
1	ALD	0	2	0	0	1.2
1	unknown	0	0	0	0	0.5
1	NAFLD	0	0	80	3	0.6

1	AIH	0	0	0	0	1.4
1	unknown	0	1	3	0	1.8
1	unknown	0	1	0	0	5
1	NAFLD	0	1	90	3	0.4
1	DILI	1	1	60	2	0.3
1	PBC	0	0	0	0	2
1	NAFLD	0	0	20	1	0.4
1	AIH	0	1	20	1	1
1	unknown	0	0	0	0	0.9
1	NAFLD	1	1	60	2	0.6
1	HBV	0	1	0	0	0.3

ALT (U/l)	yGT (U/l)
19	244
342	507
67	225
19	738
526	74
355	172
194	477
54	329
38	266
1216	429
25	15
85	33
94	31
54	120
60	181
707	197
64	97
85	130
42	85
50	38
51	535
95	469
24	146
129	21
123	33
209	447
140	1598
32	40
196	545
78	67
51	37
168	133
260	55
524	68
55	302
42	79
26	30
54	121
19	76
109	42
569	134
86	186
38	94
57	46

71	104
38	30
94	203
73	221
13	19
346	705
136	1995
80	80
120	102
49	47
30	76
204	145
908	267
18	60
321	107
94	108
9	19
21	111
21	21
107	758
47	154
52	259
218	167
148	629
146	1009
233	80
241	625
347	54
46	158
21	79
25	81
35	99
947	110
193	374
99	1188
39	127
88	425
53	42
34	69
64	313
318	131
47	141
55	357
69	49

57	115
18	58
44	307
108	32
32	162
33	26
76	148
230	334
336	353
19	288
64	180
361	123
29	151
112	1082
85	59
42	1221
50	161
84	40
66	475
66	61
41	88
101	28
131	866
54	37
238	57
33	47
121	683
30	20
54	100
50	20
2348	148
109	53
295	24
102	64
48	243
50	150
78	14
88	151
66	21
277	405
69	80
76	678
93	68
53	199
266	216
64	111
292	2070
142	31
9.8	56

259	618
135	209
487	190
156	78
6	34
322	303
111	636
45	250
10	13
13	41
36	19
